# Centroid Optimization of DNN Classification in DOA Estimation for UAV

**DOI:** 10.3390/s23052513

**Published:** 2023-02-24

**Authors:** Long Wu, Zidan Zhang, Xu Yang, Lu Xu, Shuyu Chen, Yong Zhang, Jianlong Zhang

**Affiliations:** 1 School of Computer Science and Technology, Zhejiang Sci-Tech University, Hangzhou 310018, China; 2 School of Information Science and Technology, Zhejiang Sci-Tech University, Hangzhou 310018, China; 3 Keyi College of Zhejiang Sci-Tech University, Shaoxing 312369, China; 4 Institute of Optical Target Simulation and Test Technology, Harbin Institute of Technology, Harbin 150001, China

**Keywords:** direction-of-arrival (DOA) estimation, centroid optimization, deep neural networks (DNN), multi-label classification

## Abstract

Classifications based on deep learning have been widely applied in the estimation of the direction of arrival (DOA) of signal. Due to the limited number of classes, the classification of DOA cannot satisfy the required prediction accuracy of signals from random azimuth in real applications. This paper presents a Centroid Optimization of deep neural network classification (CO-DNNC) to improve the estimation accuracy of DOA. CO-DNNC includes signal preprocessing, classification network, and Centroid Optimization. The DNN classification network adopts a convolutional neural network, including convolutional layers and fully connected layers. The Centroid Optimization takes the classified labels as the coordinates and calculates the azimuth of received signal according to the probabilities of the Softmax output. The experimental results show that CO-DNNC is capable of acquiring precise and accurate estimation of DOA, especially in the cases of low SNRs. In addition, CO-DNNC requires lower numbers of classes under the same condition of prediction accuracy and SNR, which reduces the complexity of the DNN network and saves training and processing time.

## 1. Introduction

In recent years, unmanned aerial vehicles (UAV) have gradually appeared on the frontline of disaster rescue [1]. Although video cameras have been used as embedded tools for UAVs to solve such problems [2], the auxiliary role of audio is particularly important in environments with poor visual conditions [3]. Therefore, the direction of arrival (DOA) estimation of signal sources has become one of the important research topics [4].

Many methods have been proposed for DOA estimation using array signal detection, including the multiple signal classification (MUSIC) [5] by the principle of Eigen decomposition and the estimation of Signal Parameters via Rotational Invariance Techniques (ESPRIT) [6]. Nonlinear algorithms, such as deep learning (DL), are also applied to DOA estimation [7]. Compared with DL methods, conventional DOA estimation algorithms are usually limited by many factors [8]. For example, the received coherent signal [9] causes the permeation of the signal subspace [10] and noise subspace, which lowers the accuracy of conventional DOA estimation algorithms. Ge [8] provided a systematic review of research on DOA estimation by deep neural network methods. In his research, the advantages of DL methods include detection flexibility, accurate detection, increased number of resolvable targets by a limited number of detector elements. Goodman et al. [11] found that deep learning was more robust to significant calibration errors by comparing constrained integer optimization and deep learning. Nevertheless, DL methods convert DOA estimation into pattern recognition [12]. The extracted data features help the distinction between signal and noise and improve the accuracy of DOA estimation.

In 2020, a convolutional recurrent neural network (CRNN) for DOA estimation was proposed by Küçüket et al., and implemented in Android smartphones for real-time hearing aid [13]. Xiang et al. proposed a novel feature-to-feature phase enhancement approach to reduce the phase error of received data and improve the DOA estimation accuracy [14]. In 2021, a reverberation-aware network deep-learning training method was proposed by Liu et al. The proposed method integrates the reverberation information with the input network features to alleviate the dependence of DNN on the match of environment data sample [15]. Li et al. discussed the DOA estimation based on the ResNet model for underwater acoustic arrays to extract covariance matrices in different directions [16]. Huang et al. experimentally demonstrated a machine-learning-enabled DOA estimation that relaxes the heavy reliance on complicated antenna arrays and high-cost computers [17].

Deep Neural Network Classification (DNNC) is one of the popular approaches in Deep Learning classification for DOA estimation. However, the classification approaches typically necessitate bulky-sized angular classification techniques, making them ineffective for accurate detection. This paper proposes Centroid Optimization based on Deep Neural Network Classification (CO-DNNC). The CO-DNNC algorithm consists of signal preprocessing, classification network, and Centroid Optimization. Due to the varied frequency by different people and words, the phase differences are not convenient for detection. The signal intensity is considered the input of the network. Simulation and experiments prove that CO-DNNC has high precision and accuracy. Compared with DNNC, the precision can be improved by one to three times. Especially in the case of low signal-to-noise ratio (SNR), the measurement error of CO-DNNC is only 1/2~1/4 of the measurement error of DNNC. The accuracy of CO-DNNC is two to three times the accuracy of DNNC with the same error tolerance under the condition of the same SNR and number of classes. The Centroid Optimization is flexible in the varied implementation of classification networks and helpful to performance improvement.

## 2. Theory

### 2.1. Signal Model

This paper is considering the rescue mission of UAVs, focusing on the detection of human voices in close ranges based on passive probing. The detectors are placed under the rotors of UAVs. According to the installation, the layout of the detectors is designed as a circular array to achieve 360° signal detection [18]. The circular array is centered on the mass center of the UAV. Assume that the number of array elements is *N* and the array is uniformly distributed [19]. The azimuth of the direction of the incident signal is *θ*, where *θ* ∈ [0 2π). The received signal at the nth antenna element can be given as

(1) $$r_{n}=c_{n}\cdot s+{noise}_{n}$$

Here, *c_n_* is the *n*th antenna response vector, and *n* ∈ {1…N}. *s* is the incident signal. *noise_n_* is the noise signal. The noise includes the ambient noise and inherent noise of the detector. The ambient noise received by each detector should be almost the same. However, the inherent noise of the detectors is different and independent of each other. It can be represented by the Gaussian white noise.

In the detection of human voices, the probing is taken passively. The antenna does not transmit known signals, which leads to the troublesome design for matched filters. Meanwhile, different languages, words, and speakers result in varied frequencies of voices, which make it hard to measure the phase differences of detected signals. Thereby, the power of voices is measured for azimuth detection instead of phase differences of signals. The power received by the *n*th array element can be expressed as

(2) $$P_{n}=P_{n}^{\prime }+P_{noise}$$

*P_noise_* represents the noise power. *P′_n_* is the received power and can be further denoted as

(3) $$P_{n}^{\prime }=\frac{P_{t}A_{r}}{4\pi R_{n}{}^{2}}$$

*P_t_* is the average power of the incident signal over a short period of time. *A_r_* is the effective area of the receiving antenna. *R_n_* represents the distance between the signal source and each detector. Because it is a short-distance detection, the distance differences from the signal source to different array elements cannot be ignored.

### 2.2. DNN Classification (DNNC) and Centroid Optimization of DNN Classification (CO-DNNC)

**Figure 1:**
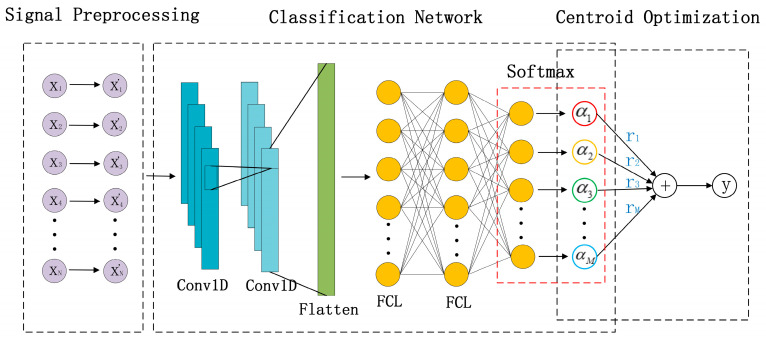
Structure of Centroid Optimization of Deep Neural Network Classification (CO-DNNC).

In Figure 1, DOA estimation based on Centroid Optimization of DNN Classification (CO-DNNC) algorithm consists of three parts: Signal preprocessing, classification network (DNNC), and Centroid Optimization.

In preprocessing, an automatic gain setting algorithm is implemented to prevent the signal intensity from being too low or too high, lowering the difficulty of network feature extraction. However, due to different ranges and intensities of incident voices, the power of the signals should be at the same level before being fed to the network. The received signal power by all detectors is also normalized.

(4) $$x_{n}=\frac{P_{n}}{\sum_{i=1}^{N}P_{i}}$$

*x_n_* is the ratio of the received signal power by the *n*th detector to the total received signal power by all array elements.

The DNNC network consists of one-dimensional convolutional layers (Conv1D) and fully connected layers (FCL). The preprocessed input data gets feature extraction by the Conv1Ds. The extracted features are reshaped from 2D to 1D and fed to FCL. In this paper, Relu is used as the activation function, and specify the binary cross-entropy as the loss function. We can train the whole network by minimizing the loss function iteratively. In the end, the probability of each class is output through Softmax. In conventional classification based on Deep Learning, the output is the probability of the existence of a signal in each direction, which is the flag to determine the predicted direction according to the highest probability. In CO-DNNC, the probabilities are fed to the Centroid Optimization.

Based on the probabilities of the existence of the signal in each direction, the Centroid Optimization of CO-DNNC estimates the azimuth of the received signal. The coordinates *r_m_* are the classified angles. The weight is the probability of each class *α_m_* [20]. The azimuth of received signal can be calculated as

(5) $$y=\sum_{m=1}^{M}r_{m}\alpha_{m}$$

## 3. Experiments

### 3.1. Datasets

Assume that the detectors are omnidirectional antennas and located under the UAV rotor. The layout of the detectors is a circular array in uniform distribution, with the mass center of the UAV as the center of the detector circle. The distance between each detector and the center is 0.5 m. Assume that the signal source is in short distance from the UAV. The distance between the source and the center of the detectors is 10.5 m. The power of received signals is calculated according to Equation (3). Since the power of the speech signal cannot maintain consistency, the ratio of signal to noise (SNR) is employed as the ratio of the mean of signal to the mean of noise, shown as Equation (6). The detected power of the signal will be the addition of the power of the received signal and noise, shown as Equation (7).

(6) $$SNR=\frac{mean({\tilde{P}}^{\prime })}{mean({\tilde{P}}_{noise})}$$

(7) $${\tilde{P}}_{n}={\tilde{P}}^{\prime }+SNR\cdot {\tilde{P}}_{noise}$$

where $\tilde{P}\prime$ is the simulated received signal and ${\tilde{P}}_{noise}$ is the simulated received noise.

**Figure 2:**
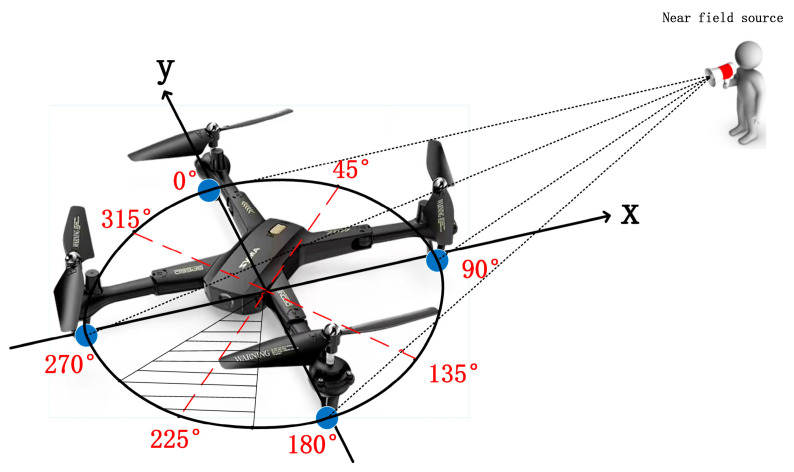
Uniform circular array of UAV and incident wave.

In the following experiments, it is assumed that the UAV has 4 detectors, shown as blue dots in Figure 2. The classification network divides the detection area 0~360° into 8 classes. The interval of each class includes 45°, shown as the shaded area in Figure 2. Any angle generated in the interval is considered to be the same class. The values of the signal and noise employed in this paper are from the IEEESPCup2019, DREGON dataset [21,22]. The clean-recordings-speech is used as the signal source, and the clean-recordings-white noise is used as the noise, including the ambient noise and inherent noise. Each pair of values corresponds to one randomly selected azimuth of incident signal. The detected powers of signal and noise are calculated by Equations (3) and (7) for the 4 detectors. The SNR is set to 10. Eight classes are considered. The azimuth of the signal source is randomly selected. A total 1600 random azimuths of the dataset are generated in each class. Eight hundred of the dataset are taken as the training set. The remaining 800 azimuths are used as the testing set. Therefore, the sizes of the training and testing sets are both 6400 × 4.

### 3.2. Performance Metrics

Accuracy (ACC) and root mean square error (RMSE) are utilized for quantifying the performance of the DOA estimation.

The root mean square error (RMSE) [23] is defined as

(8) $$RMSE=\sqrt{\frac{1}{K}\sum_{k=0}^{K-1}{\left(\theta_{k}-{\hat{\theta }}_{k}\right)}^{2}}$$

where $\theta_{k}$ is the true azimuth and ${\hat{\theta }}_{k}$ is the predicted azimuth. *K* represents the total number of signal samples.

ACC is defined as the ratio of the number of correct estimations to the number of total samples [24]. The correct estimation is defined as the estimation with an estimation error less than the tolerance. The default tolerance is half the interval between classes.

(9) $$ACC=\frac{1}{K}\cdot \sum_{i=0}^{K-1}\varepsilon (\lambda -\left|\theta_{k}\right|)$$

which ε(θ) can be expanded to

(10) $$\varepsilon (\theta )=\left\{\begin{array}{ll} 0 & \theta <0\\ 1 & \theta \geq 0 \end{array}\right.$$

### 3.3. Experiment Validation

Table 1 shows the confusion matrix of the classification network, which is DNNC here. As the proposed classification network is very simple, the accuracy of the classification is not ideal. However, the accuracy can still be above 75%.

On the other hand, the distribution of the errors is almost symmetric at the true azimuth of DOA. Meanwhile, it is less likely to generate larger errors. For example, in the case that the true azimuth of the received signal is 0°, the accuracy of classification reaches 78.5%. The probabilities of generating errors of 45° and −45° are 11.25% and 12.125%. The probabilities of generating errors of 90° and −90° are 0.5% and 0.125%. In order to demonstrate the tendency clearly, the interval of the true azimuth of DOA is set to 10°, where the true azimuths of DOA are 0°, 10°, 20°… 90°. The experiment still employs 4 detectors. The number of classes is still 8. The SNR of the testing data is set to 20 dB. The testing dataset for each true azimuth contains 800 samples. The probabilities of the classification network output are shown in Figure 2.

**Figure 3:**
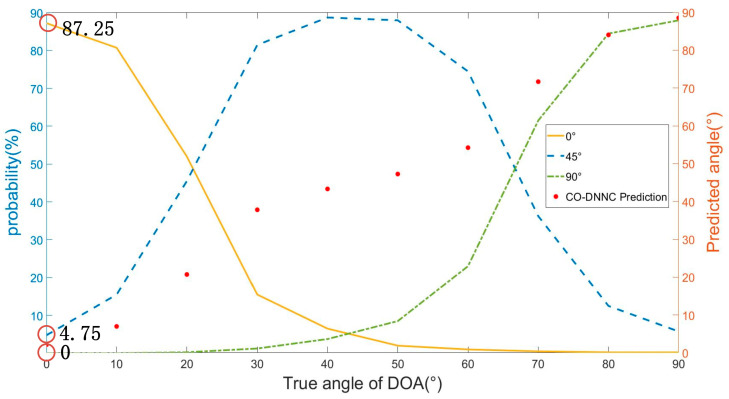
Probabilities of Softmax in DNNC and predicted azimuth by CO-DNNC.

In Figure 3, the horizontal coordinates are the true azimuths of DOA 0°, 10°, 20°… 90°. The yellow solid yellow line, blue dashed line, and green dotted line represent the probabilities of the classification network outputs on class 0°, 45°, and 90°, respectively.

In the case of the true azimuth of 0°, 87.25% of the outputs of DNNC give 0°, 4.75% of the outputs give 45°, and 0% of the outputs give 90°. The accuracy of classification is 87.25%. It seems acceptable. However, in the case of the true azimuth of 70°, 36.25% of the outputs are classified as 45°, shown in the blue dashed line, and 61.5% of the outputs are classified as 90°, shown in the green dotted line. The accuracy of DNNC is only 61.5%. The probability of wrong classification is significant, which leads to relatively great errors. The reason lies in the determination mechanism of the classification network. The classification network heavily depends on the maximum probability of the outputs without considering the gaps between the probabilities of varied classes. The data of the outputs have not been fully utilized. In Figure 3, all three lines show a symmetric shape. The probability of the output always achieves the highest value if the difference between the true azimuth and predicted azimuth is 0°. As the difference between the true azimuth and predicted azimuth increases, the probability decreases. The blue dashed line of class 45° presents the tendency completely. When the difference between the true azimuth and predicted azimuth is small, the classification network is capable of extracting distinctive features of data. As the difference grows, the data of the true azimuth possess partial features of the neighboring class. The boundary of classification turns indistinct.

Due to the symmetric shape, Centroid Optimization (CO-DNNC) is presented. In Figure 3, the red dots are the outputs of one randomly chosen sample for each true azimuth by CO-DNNC. The horizontal coordinates still represent the true azimuth of DOA. The vertical coordinates based on the right *y*-axis represent the predicted azimuth of DOA. The distribution of the red points seems approximately a straight line. The predicted azimuth of the true azimuth of 70° equals 71.6° by the Centroid Optimization. Compared with DNNC, CO-DNNC apparently has fewer errors.

**Figure 4:**
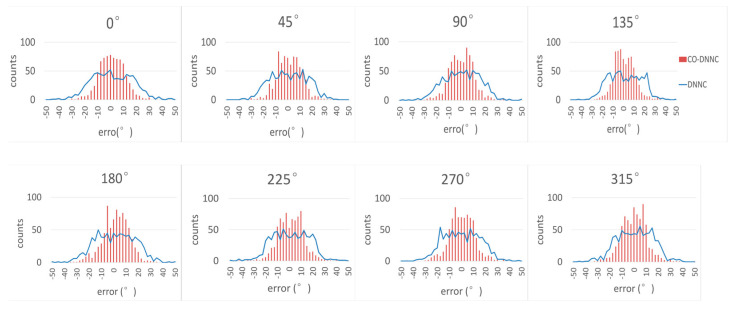
Error histogram of CO-DNNC and DNNC.

Figure 4 shows the error histograms of CO-DNNC and DNNC for all eight classes. Each class has 800 samples with random azimuths in the section. The horizontal coordinate represents the error between the true azimuth and the predicted azimuth. The vertical coordinate represents the counts of errors. The red lines represent the error histogram of CO-DNNC, and the blue curve is the one of DNNC.

The errors caused by DNNC are mostly concentrated between −20° and +20°. The errors caused by CO-DNNC are more concentrated in the range of ±10°. The RMSE of the error distribution of DNNC is 15.16° while the one of CO-DNNC is 10.20°. Therefore, the proposed CO-DNNC reduces the errors and improves the detection accuracy.

## 4. Discussion

The analysis above has shown that the mechanism of the classification network cannot either extract distinctive features or fully utilize the features, even in a situation of high SNR. When the SNR decreases, more noise is introduced, and the features of the signal are concealed, which makes it more difficult to extract the features by the classification network. Meanwhile, the interval between the classes determines the variation degrees. It will be helpful to decrease the interval and increase the number of classes to extract accurate feathers of data.

This paper investigates the impact of the SNR of signals and the number of classes on the accuracy of DOA estimation. In Table 2 and Table 3, the SNRs of the input signal are set to −20 dB, −10 dB, 0 dB, 10 dB, and 20 dB, respectively. The numbers of classes are set to 4, 8, 12, and 18, respectively. The corresponding intervals are 90°, 45°, 30°, and 20°, respectively. One thousand six hundred samples from random azimuths in each interval are taken as the training and testing datasets. Each of them has 800 samples.

### 4.1. Impact of Number of Classes and SNR

Table 2 lists the accuracies of DNNC in the cases of varied SNR and the numbers of classes. The RMSEs of the predicted DOA of CO-DNNC and DNNC are compared. In Table 2, two RMSEs are listed for each pair of SNR and the number of classes. The first number is the RMSE of DNNC and the second bold number is the RMSE of CO-DNNC.

In Table 2, the accuracy of the classification network decreases along with the increase in the number of classes for the same SNR. As more noise is introduced, the waveform of the signal is interfered with, which results in inaccurate feature extraction by the network. At the meantime, the RMSEs of CO-DNNC and DNNC increase. Taking four classes as an example where the SNR changes from −20 to 20 dB, the network accuracy decreases from 95.6% to 36.4%. The RMSE of DNNC increases from 26.9° to 95.1°, while the RMSE of CO-DNNC increases from 19.9° to 50.9°.

In Table 2, the accuracy of the classification network decreases along with the decrease of SNR for the same number of classes. As more classes are introduced, the interval between the classes shrinks, which results in the increase of the feature similarity and interference between the neighboring classes. However, it also results in the RMSEs of CO-DNNC and DNNC decrease in high SNR. For example, at 20 dB of SNR, the network accuracy can reach 81.8~95.6%, and the RMSEs of CO-DNNC and DNNC are reduced from 19.9° and 26.9° to 6.6° and 9.0°, respectively. In this case, the interference between the neighboring classes is not severe enough. The classification network can distinctively extract the data features. The shrink of the interval leads to a decrease in the RMSEs. However, when the detection situation gets worse and the SNR turns low, the interference of noise establishes supremacy over the signal. The classification accuracy is greatly reduced to 8.4~36.4%. The RMSEs depend on the intensity of noise and vary little. The RMSE of DNNC does not change much at −20 dB, along with the increase in the number of classes. However, CO-DNNC can still reduce the RMSE from 50.9° to 24.6°. All the RMSEs of CO-DNNC are lower than the RMSEs of DNNC. The differences in RMSEs between the two methods are even larger at low SNR. CO-DNNC can fully utilize the extracted features by taking the calculated probabilities as weights to estimate the DOA and reduce the error. It also shows that CO-DNNC can alleviate the interference of noise on detection. This has demonstrated that CO-DNNC is very suitable for target detection with low SNR.

In another way, in Table 2, the error of CO-DNNC with eight classes at 20 dB (10.2°) is similar to the error of DNNC with 12 classes at 20 dB (10.6°). The classification networks by deep learning algorithms are normally capable of increasing the number of classes to reduce the detection error. However, the increased classes raise the complexity of the network. CO-DNNC is capable of lowering the complexity with the satisfaction of required errors at certain SNR.

### 4.2. Impact of Tolerance

Since CO-DNNC can overcome the low SNR and reduce error, Table 2 shows the measurement accuracy in the cases of a varied number of classes and SNR. According to Equation (9), the accuracy depends on the tolerance λ. In this paper, three tolerances are set, which are λ = 50%φ, λ = 22.5%φ, and the fixed tolerance λ = 10°. φ is the interval between classes. The tolerances in λ = 50%φ and λ = 22.5%φ vary with the number of classes. λ = 50%φ represents that the tolerance is the entire interval as default tolerance, the same as the setting in the above analysis. λ = 22.5%φ represents the tolerance takes only half the interval. In practical applications, users will wisely set the parameters according to the accuracy requirements. Therefore, a fixed tolerance λ = 10° is discussed. The accuracies of DNNC and CO-DNNC (bold font) are compared in Table 3.

In Table 3, the accuracies of both CO-DNNC and DNNC are improved with the same number of classes when SNR is improved. The accuracies of DNNC and CO-DNNC increase from 18.5% and 51.4% to 88.8% and 96.5% in eight classes, respectively, when SNR increases from −20 to 20 dB and a tolerance is 50%φ. When the tolerance is fixed to 10°, the accuracies of DNNC and CO-DNNC increase from 8.4% and 24.4% to 42.4% and 70.8%, respectively.

In the cases of the same SNR, when the tolerances are set to 50%φ and 22.5%φ, the accuracies for both algorithms decrease with the increase of the number of classes. The reason is that the strategy of tolerance is set to varied tolerance according to the number of classes. The requirements for accuracy are more stringent when the interval between classes shrinks. Taking 0 dB as an example. The accuracies of DNNC and CO-DNNC drop from 76.2% and 88.7% to 24.7% and 57.5%, respectively, with a tolerance of 50%φ. The accuracies of CO-DNNC and DNNC drop from 41.8% and 66.2% to 12.5% and 29.3%, respectively, with a tolerance of 22.5%φ. However, the accuracies of DNNC and CO-DNNC improve with the increase of the number of classes when the tolerance is fixed at 10°. Taking 0 dB as an example, the accuracies of DNNC and CO-DNNC increase from 18.7% and 36.2% to 24.7% and 57.5%, respectively.

Overall, the accuracy of CO-DNNC is higher than that of DNNC. In the case of low SNR, the accuracy of CO-DNNC is significantly higher than that of DNNC. Under the circumstance of −20 dB of SNR and eight classes, the accuracy of DNNC is only 18.5% with a tolerance of 50%φ, while the accuracy of CO-DNNC reaches 51.4%. Therefore, CO-DNNC is more suitable for DOA estimation, especially under the condition of low SNR.

## 5. Conclusions

In order to improve the accuracy of DOA estimation based on classification by deep learning in random azimuths, this paper proposes a Centroid Optimization based on deep neural network classification (CO-DNNC). The received signal is calibrated in the preprocessing stage and classified by the DNN network. The output probabilities of the network are utilized to estimate the incident azimuth by the Centroid Optimization. The accuracy and precision are evaluated according to ACC and RMSE. The results show that the accuracy and precision of CO-DNNC and DNNC are both improved with the increase of SNR and the number of classes. The predicted azimuths of CO-DNNC have fewer errors than those of DNNC, especially at the condition of low SNR. Overall, the CO-DNNC measurement error is 26% to 74% of the DNNC measurement errors. The measurement accuracy of CO-DNNC is 1~3 times the accuracy of DNNC. When the SNR is low, the CO-DNNC measurement error is only 26~54% of the DNNC measurement errors. When the noise tolerance is limited at 50% of intervals, the CO-DNNC measurement accuracy is 20~33% higher than the DNNC measurement accuracy. In addition, CO-DNNC achieves the same prediction accuracy on the condition of fewer classes, which reduces the complexity of the network and the training time. The method proposed in this paper is suitable for short-distance detection. In the future, we will try to improve the performance of the array element or add phase features to get more accurate results and apply this scheme to three-dimensional space.

## Figures and Tables

**Table 1:** Confusion matrix of DNNC classification.

Predicted Azimuth of DOA	True Azimuth of DOA							
	0°	45°	90°	135°	180°	225°	270°	315°
0°	**78.5**	9.625	0.25	0	0	0	0.5	11.125
45°	11.25	**79.375**	8.625	0	0	0	0.125	0.125
90°	0.125	10.75	**78.5**	10.5	0	0	0.125	0
135°	0	0.125	10.375	**78.625**	10.625	0.25	0	0
180°	0	0	0.625	11.875	**77.0**	10.375	0.125	0
225°	0	0	0	0.25	12.25	**76.125**	10.875	0.5
270°	0.5	0.125	0	0	0.375	11.125	**75.75**	12.125
315°	12.125	0.25	0	0	0	0.25	8.75	**78.625**

**Table 2:** Accuracies of DNNC and RMSEs of CO-DNNC and DNNC with varied SNRs and classes number.

Performance Metrics		SNR									
		−20 dB		−10 dB		0 dB		10 dB		20 dB	
Accuracy (%)		36.4		54.2		76.2		89.2		95.6	
RMSE (°) of DNNC	RMSE (°) of CO-DNNC	95.1	**50.9**	70.3	**44.6**	42.3	**28.9**	30.1	**19.5**	26.9	**19.9**
Accuracy (%)		18.5		33.0		58.7		77.8		88.8	
RMSE (°) of DNNC	RMSE (°) of CO-DNNC	91.0	**30.6**	65.8	**34.8**	36.0	**27.6**	20.1	**15.1**	15.2	**10.2**
Accuracy (%)		13.0		23.6		47.6		71.4		87.3	
RMSE (°) of DNNC	RMSE (°) of CO-DNNC	87.8	**25.4**	64.2	**36.2**	34.4	**28.2**	16.7	**13.8**	10.6	**7.2**
Accuracy (%)		8.4		15.9		35.9		62.4		81.8	
RMSE (°) of DNNC	RMSE (°) of CO-DNNC	93.9	**24.6**	67.6	**38**	35.6	**30.2**	16.2	**14.7**	9.0	**6.6**

**Table 3:** Accuracies (%) for CO-DNNC and DNNC with varied SNRs and numbers of classes.

Number of Classes	λ	SNR									
		−20 dB		−10 dB		0 dB		10 dB		20 dB	
4 $\left(\varphi =90{}^{\circ}\right)$	45°	36.4	**56.3**	54.2	**71.9**	76.2	**88.7**	89.2	**97.9**	95.6	**99.6**
	22.5°	18.3	**29.0**	29.3	**42.5**	41.8	**66.2**	47.4	**77.0**	48.4	**66.3**
	10°	7.8	**12.3**	12.8	**20.3**	18.7	**36.2**	22.6	**35.5**	20.6	**26.5**
8 $\left(\varphi =45{}^{\circ}\right)$	22.5°	18.5	**51.4**	31.9	**59.0**	58.8	**68.1**	77.8	**88.0**	88.8	**96.5**
	11.25°	9.4	**27.0**	16.2	**30.8**	31.6	**42.8**	43.7	**66.0**	48.7	**78.1**
	10°	8.4	**24.4**	14.6	**27.2**	28.4	**38.7**	38.9	**62.3**	42.4	**70.8**
12 $\left(\varphi =30{}^{\circ}\right)$	15°	13.0	**44.0**	21.6	**55.8**	47.6	**53.1**	71.4	**81.3**	87.3	**94.9**
	7.5°	6.3	**23.0**	11.1	**28.6**	25.6	**30.1**	40.0	**61.1**	47.8	**80.8**
	10°	8.5	**30.2**	14.8	**38.3**	33.6	**38.9**	51.6	**69.9**	62.4	**88.3**
18 $\left(\varphi =20{}^{\circ}\right)$	10°	8.4	**31.7**	15.1	**49.9**	24.7	**57.5**	62.4	**69.0**	81.7	**90.3**
	5°	4.2	**16.2**	7.7	**26.2**	12.5	**29.3**	34.5	**47.8**	44.4	**74.7**
	10°	8.4	**31.7**	15.1	**49.9**	24.7	**57.5**	62.4	**69.0**	81.7	**90.3**

## Data Availability

The data that support the findings of this study are openly available at https://github.com/yu-zhongyang/CO-DNNC.git.
